# Diagnostic Performance of Gene Expression and dd-cfDNA in Multiorgan Transplant Recipients

**DOI:** 10.1097/TXD.0000000000001772

**Published:** 2025-02-21

**Authors:** Ziad S. Zaky, Stephanie A. Clifford, James N. Fleming

**Affiliations:** 1 Department of Kidney Medicine, Cleveland Clinic, Cleveland, OH.; 2 Medical Affairs, Transplant Genomics, Inc, Framingham, MA.

## Abstract

**Background.:**

The aim of this analysis was to evaluate early signals of the utility of gene expression profile (GEP) and donor-derived cell-free DNA (dd-cfDNA) for ruling out subclinical rejection in multiorgan transplant recipients.

**Methods.:**

This was a prospective, single-center, observational pilot study that began enrolling patients in September 2022. Participants were enrolled after providing informed consent and had biomarker samples drawn before surveillance or for-cause biopsy. GEP result of TX was considered negative and a dd-cfDNA of ≤0.69% was considered negative, regardless of a nonrenal organ.

**Results.:**

There were 49 participants with 55 surveillance and/or for-cause biopsies. After exclusion of biopsies not paired with biomarkers, 51 biopsies were evaluated with at least 1 biomarker. Fifty-one biopsies had paired GEP results, whereas 47 biopsies had paired dd-cfDNA results. Overall, there were 12 biopsy-proven acute rejections (24%), 5 of which were T cell–mediated rejections (4-1A and 1-1B), 2 were antibody-mediated rejections, and 5 were borderline for T cell–mediated rejections. GEP by itself in 51 biopsies demonstrated a sensitivity of 17%, specificity of 74%, positive predictive value of 17%, negative predictive value of 74%, and balanced accuracy of 61%. Among 47 paired biopsies, dd-cfDNA demonstrated a sensitivity of 67% and specificity of 37%. Median dd-cfDNA was above the positivity threshold for both participants with rejection on biopsy and without (1.86% versus 1.35%, respectively). When evaluating GEP, specifically in surveillance biopsies and patients with liver transplants, diagnostic performance was maintained.

**Conclusions.:**

In this pilot analysis, GEP maintained a high negative predictive value in a multiorgan cohort, regardless of the nonrenal organ. dd-cfDNA did not have good performance when using the kidney threshold cutoff, which was expected and driven by the liver component of multiorgan recipients. Further technological advances with dd-cfDNA to differentiate organs and multiple donors could be impactful. The results support the use of GEP for ruling out kidney rejection in a multiorgan population, including those with a liver transplant. Further evaluation is necessary to confirm the results.

Rejection of a transplanted organ continues to be a significant concern for kidney transplant recipients (KTRs) and their medical providers. There have been advancements over the years that have improved short-term outcomes and the incidence of rejection, such as improvements in immunosuppression medications and HLA matching.^1,2^ Despite these advancements, immune-mediated graft inflammation and rejection remain a high concern, with clinical acute rejection occurring in 10%–15% of patients within the first year posttransplantation.^1^ Furthermore, among patients with death-censored allograft failure, rejection remains the predominant cause of graft loss.^3,4^ Subclinical acute rejection has been reported to occur in 20%–25% of patients in the first 12–24 mo posttransplant.^5^ However, subclinical inflammation (borderline changes on histopathology that fall below the diagnostic threshold for TCMR, grade IA based on Banff classification) has been reported to occur in up to 40% of KTRs during the first year posttransplant.^5^ The incidence of both subclinical acute rejection and subclinical inflammation is important to note, given that both have demonstrated worse outcomes, including subsequent clinical rejection, allograft failure, and death-censored graft loss when compared with patients with no subclinical inflammation.^6-8^ The incidence of rejection in multiorgan transplant (MOT) recipients, including kidney, is not as well documented.

In 2021, 24 667 patients received a kidney transplant in the United States. Of those, 1943 patients (7.8%) received a kidney with an additional organ, being considered a MOT recipient.^9^ This number does not include the additional patients receiving an organ transplant after initial transplantation of a different organ. As a small subset of annual organ transplants, multiorgan recipients are poorly represented in clinical outcomes research and represent clinical management challenges outside of those encountered in solitary kidney transplants. Molecular biomarkers, such as donor-derived cell-free DNA (dd-cfDNA) and gene expression profile (GEP), are increasingly being used in the management of KTRs as a noninvasive assessment for ruling out rejection; however, they are poorly studied in the multiorgan population. Providers of KTRs with additional donated organs still have creatinine, immunosuppression levels, and HLA donor specific antibodies (DSA) titers to monitor patients; however, these are insensitive and nonspecific measurements to evaluate rejection.^10^ To date, there is no clinically available noninvasive biomarker to monitor for renal allograft rejection in MOT recipients. There have been investigations of both dd-cfDNA and GEP to evaluate the rejection of a cardiac allograft in MOT (dual organ) recipients. These data are limited yet encouraging that kidney-specific GEP used to evaluate renal rejection in MOT recipients may be effective.^11-13^

Currently, both GEP and dd-cfDNA are clinically available for kidney transplant patients. Both GEP and dd-cfDNA have been shown to be reliable “rule-out” tests for rejection in KTRs.^10,14-18^ However, the validity and utility of these tests in monitoring for renal allograft rejections in the setting of MOTs has yet to be investigated. The aim of this study is to evaluate the utility of GEP and dd-cfDNA as noninvasive biomarkers for renal allograft rejection in MOT recipients.

## MATERIALS AND METHODS

### Study Design

This is a prospective, single-center, observational pilot study to evaluate the performance of GEP and dd-cfDNA in ruling out clinical and/or subclinical acute rejection of the kidney in MOT recipients. Institutional review board approval and patient informed consent were obtained before enrollment. The study was conducted in abidance by the Declaration of Helsinki. Patient enrollment occurred between September 2022 and November 2023. Any patient with a simultaneous pancreas-kidney transplant, combined kidney plus other solid organ transplant, or kidney transplant after prior nonrenal solid organ transplant was eligible. Participants were not eligible to participate in the study if they were pregnant, received a transplant from an identical twin, or received a blood transfusion within the past 30 d of the study blood draw. Biomarker samples were drawn on all patients on the same day and before kidney biopsy. Surveillance biopsies were performed at 3–6 mo and 12 mo posttransplant according to our center protocol. For-cause biopsies were performed in the setting of elevated creatinine by at least 20% from nadir creatinine posttransplant after ruling out other etiologies (eg, dehydration, supratherapeutic calcineurin inhibitors levels).

### Study Objectives

The aim of this study is to evaluate the diagnostic performance of GEP as well as dd-cfDNA at the time of kidney transplant biopsy in MOT recipients.

### Biomarker Assays

Blood samples for the dd-cfDNA and GEP assays were drawn and sent to Eurofins Transplant Genomics for processing according to their collection protocol. The dd-cfDNA samples were processed using next-generation sequencing and recipient genotyping. Data were mapped to reference genomes and analyzed for percentage dd-cfDNA by a custom bioinformatics pipeline. The dd-cfDNA results were provided as a percentage of the donor-derived fraction as compared with total cfDNA by using panels of single-nucleotide polymorphisms to differentiate between donor and recipient cfDNA without requiring knowledge of donor genotypes. The transplant rejection allograft check (TRAC) assay (Eurofins Transplant Genomics, Lenexa, KS) reports the fraction of donor-derived cfDNA as a percentage with ≥0.7% being considered positive.

Blood samples for the GEP assay were processed using reverse transcriptase polymerase chain reaction and microfluidics on the Fluidigm Biomark HDTM system (Fluidigm, South San Francisco, CA). The GEPs were analyzed with the TruGraf algorithm, a gene expression algorithm analyzing the differential expression of >100 gene signatures that look at a variety of both immunologic (majority) and nonimmunologic gene expression pathways. The TruGraf assay (EurofinsTransplant Genomics, Lenexa, KS) has a previously defined probability threshold of 0.5 to differentiate the transplant excellence (immune quiescence) from the not transplant excellence phenotype (not immune quiescent).

### Study Definitions

Biopsies were classified on the basis of Banff 2019 criteria.^19^ Borderline rejection and all grades TCMR and antibody-mediated rejection (AMR) were considered positive biopsies.

### Statistical Analysis

Diagnostic performance characteristics were conducted to determine the ability of both TruGraf GEP and Viracor TRAC dd-cfDNA to accurately rule out rejection. Copper-Pearson 95% confidence intervals (CIs) were used for sensitivity, specificity, and accuracy; standard logit CIs were used for negative predictive value (NPV) and positive predictive value. Prevalence was determined using the data set available.

## RESULTS

In total, there were 49 participants enrolled who underwent 54 biopsies. Biopsies that were not paired with at least 1 biomarker were excluded from analysis, resulting in 51 paired biomarker and biopsy samples. All 51 biopsies had paired GEP results reported, whereas 47 biopsies had paired dd-cfDNA results reported. Combined liver-kidney transplant, 19 (37%) made up most of the study population. Complete population demographics are reported in Table 1. There was a total of 12 (24%) biopsy-proven acute rejection episodes, 5 of which were T cell–mediated rejection (TCMR; 4 where TCMR 1A, 1 was TCMR 1B), 2 biopsies demonstrated AMR, and 5 biopsies were considered borderline for TCMR. There were 4 circumstances where there was a rejection from the nonrenal organ in proximity to the renal biopsy; 1 patient had a positive liver biopsy approximately 1 mo before their negative kidney biopsy (GEP TX, dd-cfDNA 2.56%) and 3 participants had 1R on heart biopsies between 3 mo before and 1 mo after the kidney biopsy. All kidney biopsies were negative, and only 1 GEP was positive, whereas all dd-cfDNA tests were below the positivity threshold for kidney. In addition, there were no cases of clinically significant cytomegalovirus infection at the time of biopsy. There were 7 biopsies performed in the presence of BK DNAemia, although 6 of these polymerase chain reaction results were <500 copies/mL. One biopsy in a combined heart-kidney transplant recipient had 20 400 copies/mL and class 2 polyomavirus nephropathy on biopsy. In this case, both dd-cfDNA and GEP resulted as negative and there was no rejection noted on the biopsy.

**TABLE 1.** Demographics

Demographics	N = 49
Age, y	51 ± 12
Sex, male	28 (57%)
African American/Black	3 (6%)
Class I DSA	3 (6%)
Class II DSA	11 (22%)
Days post kidney transplant	346 (153–568)
Organs	
Combined liver-kidney	19 (39%)
Combined heart-kidney	8 (16%)
Simultaneous pancreas-kidney	6 (12%)
Kidney after liver	13 (27%)
Kidney after heart	3 (6%)
Induction agent	
IL-2RA	22 (45%)
Antithymocyte globulin	27 (55%)
Maintenance regimen	
Tacrolimus + MPA + prednisone	38 (78%)
Cyclosporine + MPA + prednisone	3 (6%)
Cyclosporine + everolimus + prednisone	1 (2%)
Tacrolimus + everolimus + prednisone	2 (4%)
Tacrolimus + prednisone	5 (10%)
Demographics of paired biopsy samples	N = 51
Organs	
Combined liver-kidney	19 (37%)
Combined heart-kidney	8 (16%)
Simultaneous pancreas-kidney	7 (14%)
Kidney after liver	14 (27%)
Kidney after heart	3 (6%)
For-cause biopsy, n (%)	24 (47%)
SrCr mg/dL	1.36 (1.13–1.90)

DSA, donor specific antibodies; IL-2RA, interleukin-2 receptor antagonist; MPA, mycophenolic acid; SrCr, serum creatinine.

**FIGURE 1.**
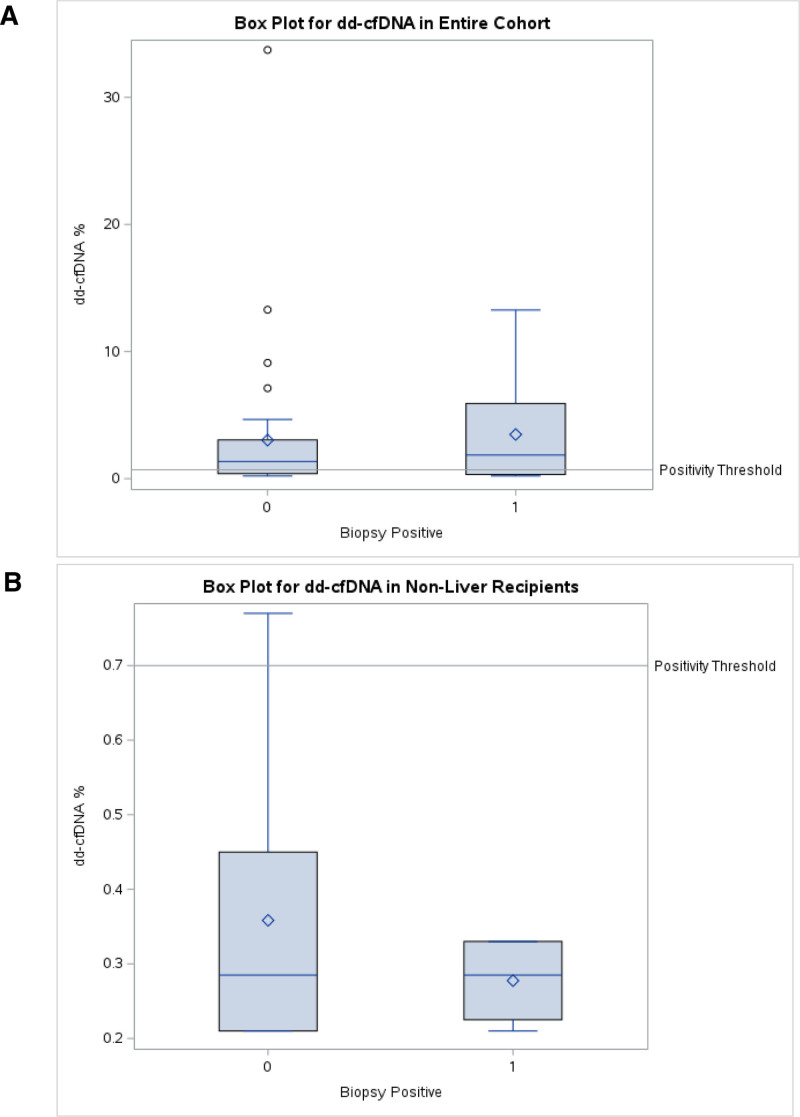
dd-cfDNA values compared with diagnostic threshold in entire cohort (A) and in non–liver recipients only (B). dd-cfDNA, donor-derived cell-free DNA.

Diagnostic performance of GEP was evaluated individually among 51 paired biopsies. This GEP demonstrated a low sensitivity of 17% and positive predictive value of 17%, with a much higher specificity of 74% and NPV of 74%, and a balanced accuracy of 61% (Table 2). The diagnostic performance of dd-cfDNA was then assessed among 47 paired biopsy samples, which demonstrated a sensitivity of 67% along with a low specificity of 37%. Median dd-cfDNA was above the positivity threshold for both participants with rejection on biopsy and without (1.86% [0.33–5.92] versus 1.35% [0.40–3.05], respectively, when evaluating the entire cohort; Figure 1A). It is noteworthy that 29 of 31 dd-cfDNA-paired biopsies with a liver transplant had a positive dd-cfDNA (median 1.68% [0.88–3.65]), with only 8 episodes of biopsy-proven acute rejection. This pattern of dd-cfDNA is expected because of the size of the liver contributing to a higher percentage of circulating dd-cfDNA.^20^ When evaluating only non–liver multiorgans, the median dd-cfDNA was 0.29% (0.21–0.45) in biopsies negative for rejection and 0.29% (0.23–0.33) in biopsies that were positive for rejection (Figure 1B). Notably, there were no dd-cfDNA values above the positivity threshold in positive-biopsy paired samples and 1 result above the positivity threshold in the negative-biopsy paired samples (0.77%).

**TABLE 2.** Diagnostic performance of peripheral blood GEP and dd-cfDNA

	GEP (N = 51)	dd-cfDNA (N = 47)
Sensitivity	16.7% (2.1%-48.4%)	66.7% (34.9-90.1)
Specificity	74.4% (57.9%-87.0%)	37.1% (21.5-55.1)
PPV	16.7% (4.8%-44.1%)	26.7% (18.5-36.9)
NPV	74.4% (68.0%-79.9%)	76.5% (56.7-89.0)
Balanced accuracy	60.8% (46.1%-74.2%)	44.7% (30.2-59.9)

dd-cfDNA, donor-derived cell-free DNA; GEP, gene expression profile; NPV, negative predictive value; PPV, positive predictive value.

As subanalyses, GEP diagnostic performance was assessed specifically in surveillance biopsies, as this is the context for which the test was developed and in participants with a liver transplant. GEP performance was excellent in surveillance biopsy contexts, with a specificity of 80%, NPV of 91%, and accuracy of 75% (Table 3). In 33 participants with a transplanted liver, GEP had similar diagnostic performance as to the entire population, with a specificity of 84%, NPV of 75%, and balanced accuracy of 67% (Table 4).

**TABLE 3.** Diagnostic performance of peripheral blood gene expression profile in surveillance biopsies

	N = 28
Sensitivity	33.3% (0.8%-90.6%)
Specificity	80.0% (59.3%-93.2%)
PPV	16.7% (3.3%-54.3%)
NPV	90.9% (81.4%-95.8%)
Balanced accuracy	75.0% (55.1%-89.3%)

NPV, negative predictive value; PPV, positive predictive value.

**TABLE 4.** Diagnostic performance of peripheral blood gene expression profile for participants with a transplanted liver

	(n = 33)
Sensitivity	12.5% (0.3%-52.7%)
Specificity	84.0% (63.9%-95.5%)
PPV	20.0% (3.1%-65.8%)
NPV	75.0% (68.7%-80.4%)
Balanced accuracy	66.7% (48.2%-82.0%)

NPV, negative predictive value; PPV, positive predictive value.

## DISCUSSION

In this pilot analysis, TruGraf GEP maintained a high NPV in ruling out renal rejection in MOT recipients, regardless of whether the nonrenal organ was additionally transplanted simultaneously, combined, or secondary after a prior nonrenal solid organ transplant. This is an important finding because medical providers of these patients have only been able to use conventional diagnostics and biopsy to assess for rejection. To date, there have been no publications supporting the use of GEP in assessing renal rejection in MOT recipients; however, the notion has been supported by research in cardiac allograft rejection assessment in dual organ transplant recipients (although this is limited).^11,12^

TruGraf GEP is a blood assay that was developed and validated to detect the absence of clinical rejection in KTRs. The patient’s pattern of gene expression up and downregulation is compared with the GEPs of 2 reference populations to differentiate the TX (normal, no rejection) from the non-TX phenotype (including subclinical rejection). In theory, because this test was developed specifically to evaluate kidney transplant rejection, it should be useful in detecting KT rejection in MOT recipients as well, as this had just never been studied before. In this study, the NPV of TruGraf GEP was 74.4% (95% CI, 68.0-79.9), which is similar to reported TruGraf GEP kidney diagnostic performance in single KTRs with a range of 78%–88%.^10,14^ Specifically in the context of surveillance biopsies, the NPV of TruGraf GEP was 90.9%, which is a more in-line comparison with previous analyses.

As expected, TRAC Kidney (dd-cfDNA) did not have good performance when using the kidney diagnostic threshold cutoff. The test is contraindicated to use in multiple donors due to its percentage measurement of cfDNA, that is, MOT recipients will have a higher “acceptable” level of circulating dd-cfDNA simply due to having more donor-derived physiologic cfDNA present at baseline. This is especially pronounced in MOT recipients with liver or lung as these organs have been identified to have a much higher baseline and variability of dd-cfDNA due to their size, which is seen in this study with liver recipients as well as other reported findings in heart-liver and heart-lung MOT.^11,20,21^ Currently, dd-cfDNA is unable to discreetly identify kidneys (or any specific organ) from other organ cfDNA; however, further technological advances to differentiate organs and multiple donors could be impactful. There are interesting developments in using methylation-based cfDNA assays in the future to achieve this.^22^ However, at this time, our findings suggest that dd-cfDNA should not be used to rule out renal rejection in MOT recipients.

Due to the rippling effects of organ allocation changes, MOTs involving a kidney have increased significantly during the past 20 y.^23^ As the technology in the field of transplant continues to advance, MOT recipients will continue to represent a significant and unique cohort of patients. The prevalence of MOT recipients will possibly continue to increase as technological advancements may translate to improved graft and overall survival. This analysis presents an expanded population that TruGraf GEP may be able to be used in. In this small sample, there was a high proportion of cellular rejection (5) and borderline (5) and 2 AMR. The current small sample size is a limiting factor; however, a larger follow-up study focusing on combined liver-kidney and heart-kidney recipients is now underway to confirm results and is expected to capture more episodes of different types of rejection. Additional considerations for future investigations include expanding to a multicenter study design, as well as including comparison of GEP scores between MOT recipients and single KTR in the future. It would also be important to evaluate the relative change in dd-cfDNA from baseline in future analyses. Nonetheless, the NPV performance reported is similar for MOT recipients in this study compared with prior reports in a single KTR. TruGraf Kidney GEP shows potential promise in being an excellent surveillance tool in ruling out kidney rejection in MOT recipients.
